# Extreme rainfall events pulse substantial nutrients and sediments from terrestrial to nearshore coastal communities: a case study from French Polynesia

**DOI:** 10.1038/s41598-020-59807-5

**Published:** 2020-02-19

**Authors:** Caitlin R. Fong, Camille J. Gaynus, Robert C. Carpenter

**Affiliations:** 1 California State University Northridge, Department of Biology, Los Angeles, USA; 2 University of California Los Angeles, Department of Ecology and Evolutionary Biology, Los Angeles, USA

**Keywords:** Ecology, Climate-change ecology, Ecosystem ecology, Tropical ecology, Environmental impact

## Abstract

Rainfall mobilizes and transports anthropogenic sources of sediments and nutrients from terrestrial to coastal marine ecosystems, and episodic but extreme rainfall may drive high fluxes to marine communities. Between January 13^th^ and January 22^nd^, 2017, the South Pacific Island of Moorea, French Polynesia experienced an extreme rainfall event. ~57 cm of rain was delivered over a 10-day storm. We quantified pulsed sediments and nutrients transported to nearshore reefs. We determined the spatial and temporal extent of the sediment pulse with estimates of water transparency. We quantified pulsed nutrients at multiple spatial and temporal scales. To determine if terrestrial nutrients were incorporated into the benthic community, we collected macroalgae over 10 days following the storm and measured tissue nutrient concentrations and δN^15^. Pulsed sediments impacted water clarity for 6 days following the storm, with greatest impacts closest to the river mouth. Nitrite +nitrate concentrations were >100 times the average while phosphate was >25 times average. Macroalgal tissue nutrients were elevated, and δN^15^ implicates sewage as the source, demonstrating transported nutrients were transferred to producer communities. Future climate change predictions suggest extreme rainfall will become more common in this system, necessitating research on these pulses and their ramifications on marine communities.

## Introduction

Riverine transport is the primary conveyance of terrestrial anthropogenic impacts to nearshore coastal communities, and these pathways are influenced by both rainfall and land use patterns^1–3^. Transport of nutrients and sediments from rivers to oceans have been documented world-wide and is a major management concern as nearshore coastal communities provide a diversity of goods and services^3–8^. Changes in land use can drive fluxes of nutrients and sediments to coastal communities^5,6^. For example, deforestation mobilizes sediment, increasing transport via rivers and runoff with rainfall events, and researchers project impacts of sedimentation outweigh that of climate change to Madagascar’s coral reefs^7^. Additionally, agricultural development has increased both the global availability and mobility of nitrogen, which can be discharged by rivers^5,6,9^. Finally, urbanization can change infiltration rates and thus inputs into rivers; thus, areas with increased urbanization experience higher discharge variance^10^. Consequently, changes in land use patterns can alter transport of nutrients and sediments from rivers to oceans.

Episodic but extreme rainfall events can create pulsed riverine discharge events, increasing both nutrients and sediments, but are challenging for systematic water quality sampling protocols to capture. We define press events as low magnitude, high frequency, long duration events and pulse events as high magnitude, low frequency, and short duration events *sensu*^11^. Extreme rainfall events can increase the discharge of nutrients and sediments into nearshore communities, creating pulsed events distinct from regular discharge^5,12^. These pulsed versus pressed subsidies probably have different impacts on coastal communities^11,13^. However, these extreme rainfall events likely are not captured by regular water sampling protocols, which typically occur at regularly scheduled intervals, over the course of weeks to months (e.g.^14^). Thus, understanding the flow of materials from terrestrial to marine systems during these episodic events is important, yet underexplored.

Coral reefs are diverse and highly productive near shore communities that may be threatened by pulsed riverine discharge events driven by episodic extreme rainfall events. Coral reefs provide a diversity of goods and services, including but not limited to pharmaceutical materials, organisms for aquaria trade, fisheries, ecotourism, generation of coral sand, protection from storm events, and cultural value, all derived from the complex biogenic structure that provides resources for reef organisms^15^. Low nutrient concentrations and low sedimentation rates are characteristic of healthy reefs^6,16,17^. However, riverine discharge can increase both nutrient and sediment transport from terrestrial to coral reef communities, and extreme rainfall events likely result in pulsed transport of these materials.

Research across multiple reefs indicates substantial pulses of substantial nutrients and sediments discharged from rivers into the water column in the form of plumes, often coupled with rainfall events. Research in Australia shows riverine discharge is the largest source of nutrients to the Great Barrier Reef Lagoon^5^. These inputs are elevated in the wet season when rainfall can generate a large flood plume^18^, likely from mobilized terrestrial sediments and resuspended sediments^19–21^. Remote sensing in the Great Barrier Reef indicates flood plume water first drives increases in suspended sediments, followed by a phytoplankton bloom and then elevated detrital matter^22^. In Hawaii, nutrient runoff driven by rainfall events drives phytoplankton blooms, though effects only last 3–8 days due to high flushing rates^23^. In Curaçao, researchers have documented river discharge nutrient pulses following rainfall events^12^. Taken together, these studies show rain driven flood events elevate nutrient and sediment concentrations in the water column, which may have substantial impacts on coral reef benthic communities. Thus, pulsed nutrient and sediment events from terrestrial communities to coral reefs are common, and changes to the water column have been documented globally, yet uptake of nutrients by the benthos following these events remains understudied.

**Figure 1:**
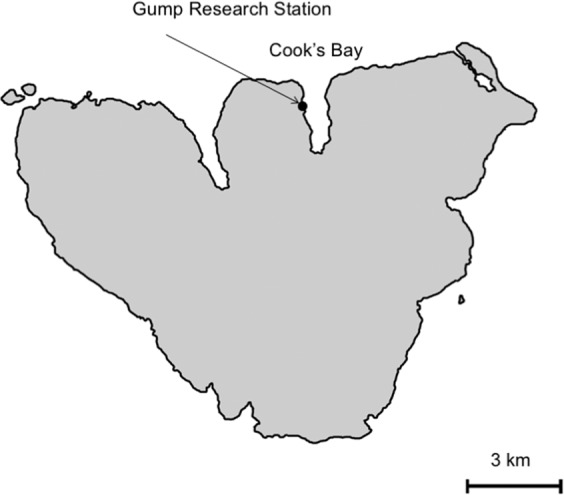
Map of Moorea.

To understand transport and transference of materials from terrestrial to coral reef communities, we quantified inputs of nutrients and sediments to Cook’s Bay in Moorea, French Polynesia following an extreme rainfall event (Fig. 1).

## Results

### Rainfall

**Figure 2:**
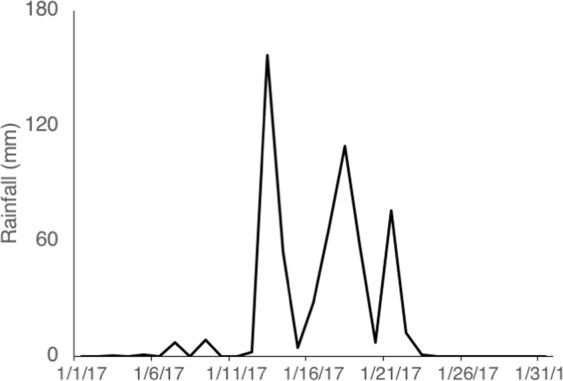
Total daily rainfall (mm) from 1 January 2017 to 31 January 2017.

Between January 13^th^ and January 22^nd^ 2017, the Gump weather station recorded ~57 cm of rain over a 10-day storm (Fig. 2). This was an extreme rainfall event for this location; Gump Research Station has conducted continuous rainfall monitoring from August 6, 2006 and in that time period, January 13, 2017 was the 2^nd^ rainiest day while January 18, 2017 was the 11^th^ rainiest day on record (maximum on March 5, 2010 at 21.5 cm of rain).

### Sediment pulse

**Figure 3:**
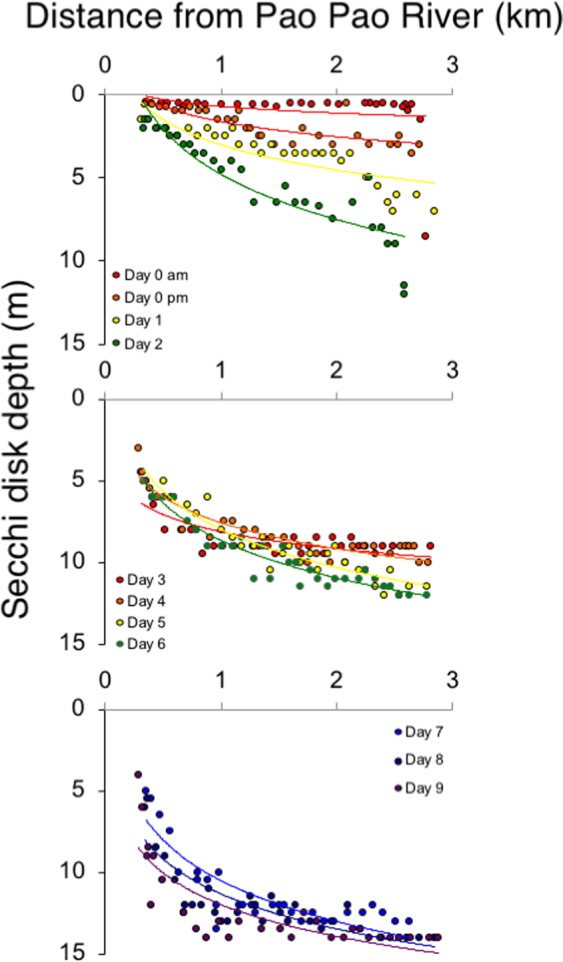
Relationship between secchi disk depth (m) and distance from Pao Pao river after a major storm event. Secchi disk depth was measured the day after in the morning and afternoon, and then every day through 9 days after the storm.

Rainfall pulsed sediment into nearshore waters for at least 1 week following the rainfall event (Fig. 3). Over our entire sampling period, secchi disk depth was shallowest closest to the mouth and deepest farthest from the mouth, although both the amount of, and the gradient in suspended sediment varied spatially and temporally following the storm. The morning of the last day of rain, there were no strong spatial patterns in water clarity, and a plume of suspended sediments extended close to 3 km from the mouth of the river. However, 4 hours later, water clarity had begun to improve starting around 1 km from the mouth of the river. One day after the storm, secchi disk depth more than doubled, suggesting sediment and suspended particulate matter had begun to either settle or was moved out of the bay. This trend continued through day 2, with points farther from the mouth of the river improving more rapidly. Water transparency continued to improve through day 6, although secchi disk depth remained less than 12 m, even at the furthest distances from the mouth of the river. By days 7 and 8 after the rainfall, secchi disk depth continued to increase to 14 m at the farthest distance from the river mouth. By the final day of sampling^9^, secchi disk reach maximal depths (14 m) less than 1 km from the river mouth, which was equal to secchi disk depth at the farthest point from the river.

On average, suspended sediment had a total nitrogen content of 0.34 ± SE 0.02%.

### Nutrient pulse

**Figure 4:**
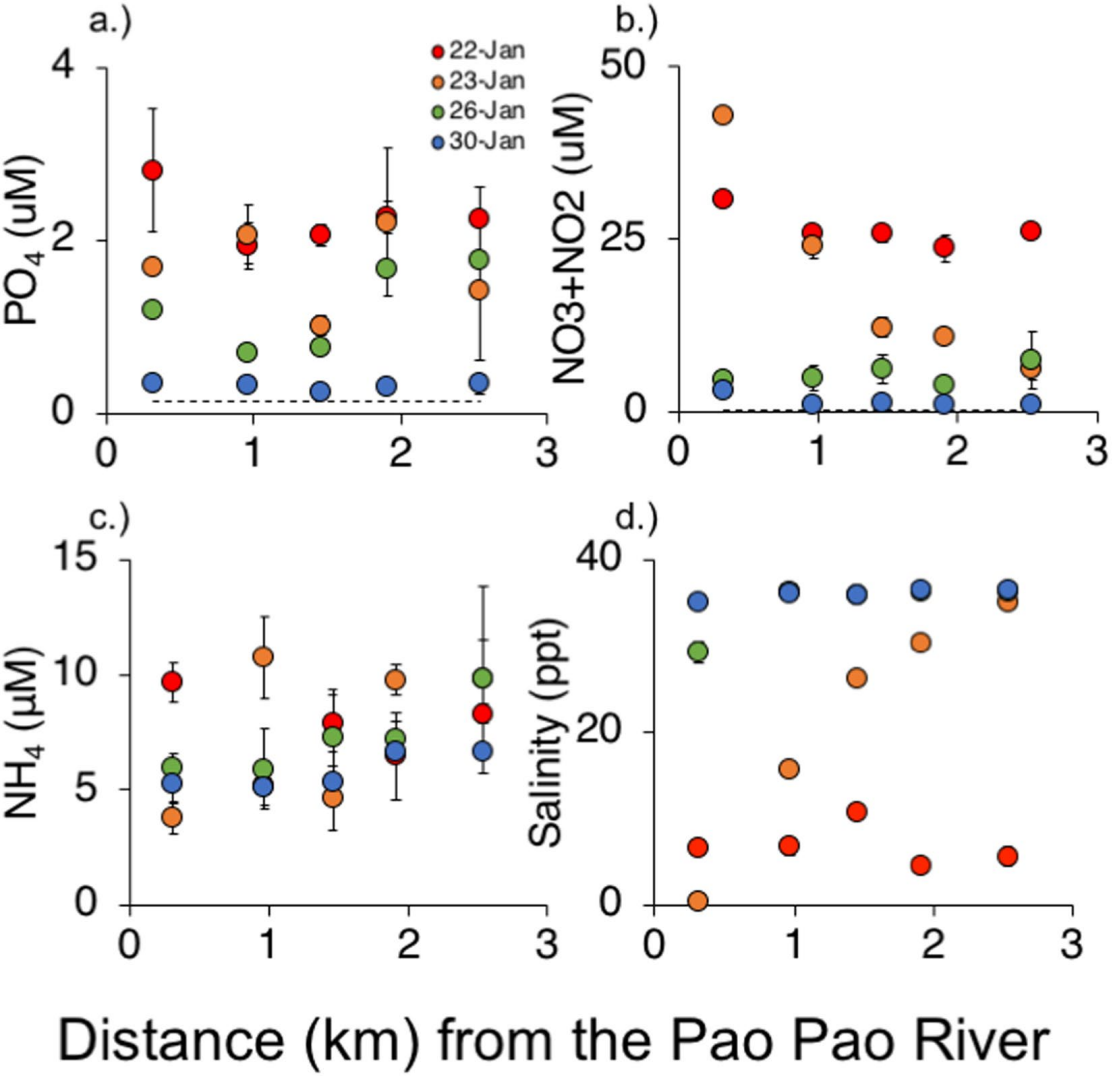
Concentrations of phosphate (**a**), nitrite + nitrate (**b**), ammonia (**c**) as well as salinity (**d**) (mean +/− SE). Samples were collected 0, 1, 4, and 9 days after a storm event at multiple distances from the mouth of the Pao Pao River. Dashed lines in panels (a,b) are average concentrations measured by the MCR LTER.

Over our sampling period, phosphate (PO_4_) concentrations were elevated and ranged from 0.19 to 4.15 μM (Fig. 4a); in contrast, the typical concentration of phosphate in Cook’s Bay is 0.15 ± 0.01 μM PO_4_. On the final day of rain, we found phosphate concentration was highest closest to the mouth of the river, with concentrations reduced by ~20% at the mouth of the bay. One day after the final rainfall, phosphate remained elevated but spatially variable, averaging 1.68 ± SE 0.22 μM PO_4_. By day 4, concentrations were highest farthest away from the mouth at 1.77 ± SE 0.79, suggesting phosphate had moved down the bay. On day 9, phosphate remained elevated, but with no strong spatial pattern with an average of 0.32 ± SE 0.02 μM PO_4_ across sites.

We found limited spatial and temporal patterns in NH_4_ concentrations, which averaged 6.86 ± SE 0.46 μM across all samples (Fig. 4b); while NH_4_ is not routinely sampled by the LTER, NH_4_ concentrations in the lagoon of nearby Tikehau Atoll averaged 1.9^24^.

In contrast, we found strong spatial and temporal patterns of NO_3_ + NO_2_ concentrations. NO_3_ + NO_2_ ranged over our sampling period from 0.44–46.60 μM (Fig. 4c); in contrast, typical concentrations are 0.40 ± SE 0.06 μM. The first day after the storm, NO_3_ + NO_2_ concentrations were elevated and ranged from 23–30 μM, and decreased marginally with distance from the mouth of the river. Day 1 had the strongest spatial patterns in NO_3_ + NO_2_ concentrations, with the highest concentration at the mouth ( >40 μM) and concentration lowest at the mouth of the bay (~2.5 μM). By day 4, this spatial pattern had mostly dissipated but concentrations remained elevated at ~5.4 μM down the bay. By day 9, we found relatively low levels of NO_3_ + NO_2_ from the mouth of the river through the end of the bay, averaged ~1.5 μM across all samples.

On the last day of the storm, there was a lower salinity water layer at the top with little mixing out the pass (Fig. 4d). Down the bay, salinity was 6.9 ± SE 0.65 ppm. On the first day after rain, salinity increased from ~0 to ~35 as the distance from the mouth of the river increased from 0.3 to 2.5 km, providing evidence of mixing. By day 4, fresh water was detectable only at the mouth and by day 9, salinity was ~35 from the mouth of the river through the pass, suggesting that the period of the higher discharge event had passed.

### Macroalgal tissue nutrients

**Figure 5:**
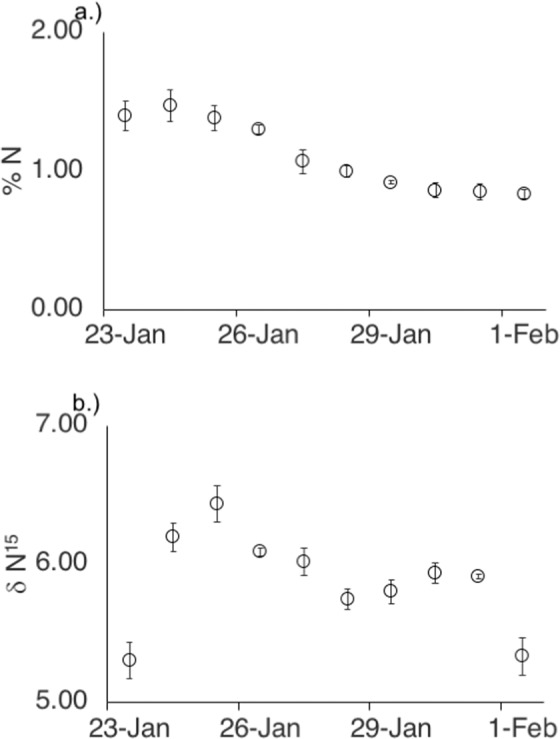
Tissue nitrogen percentage as well as δN^15^ in *Padina boryana* on days 1 through 10 after the storm event.

Rainfall elevated tissue N in *Padina boryana* collected from Gump Reef (Fig. 5a). Percent N was highest immediately after the storm and then declined over the 10-day sampling period. Between days 1 and 10, tissue N was reduced by ~40% from 1.39 ± SE 0.10% to 0.83 ± SE 0.03%.

Rainfall also may have elevated δN^15^ ratios in *P. boryana* tissue (Fig. 5b). δN^15^ increased ~20% between the first and third day after the storm, from 5.31 ± SE 0.13 to 6.44 ± SE 0.13 suggesting a change in enrichment source. After the fourth day, δN^15^ levels decreased and by the end of the sampling period, δN^15^ levels had returned to the same level as the first day after the storm.

## Discussion

Extreme rainfall was correlated with increased riverine transport of nutrients and sediments, two important drivers of nearshore community structure^6,25,26^. We documented a high magnitude, low frequency, short duration increases in transport and transference, or assimilation, of nutrients following an extreme rainfall event in Moorea, French Polynesia. Nitrite + nitrate concentrations ranged from 1–43 μM; more than 100 times higher than average conditions measured by the Mo’orea LTER since 2005^27^. Similarly, over the course of the storm, phosphate ranged from 0.25–2.8, which is 27 times higher than average conditions measured by the Mo’orea LTER since 2005^27^. These pulsed inputs were of short duration, and were either flushed out of the bay or taken up biotically after 9 days. These pulsed concentrations are also higher than concentrations documented in rivers transporting materials in Australia, Hawaii, and Curaçao^12,19,22,23^. We also document either riverine transport of sediments or resuspension of sediments associated with this rainfall event. Suspended sediments were elevated following the storm, and detected over a broad area before exiting the system either by transport or settlement. This development of a spatially broad and temporally distinct sediment plume is similar to plumes developed in other systems^5,18–20,22,23^. Both nutrients and sediments are important drivers of dynamics in a variety of nearshore coastal communities^1^, necessitating further documentation of these transport and/or resuspension events.

Pulsed nutrient and sediment additions may be one mechanism by which macroalgae can proliferate on reefs in spite of typically low nutrient and sediment availability. This finding was supported by elevated tissue nutrient concentrations following the pulsed rainfall event, indicating macroalgae in the benthic community were able to rapidly assimilate transported nutrients. Further, storms increase suspended sediments in the water, which were either transported or resuspended. These red sediments were particularly rich in nitrogen, suggesting they were transported nitrogen from terrestrial to nearshore communities. Thus, nutrients were both transported and transferred from terrestrial to marine communities. Previous research indicates pulsed nutrient supplies change the outcome of competition among macroalgal species on reefs^13^ and may shift communities to species with rapid uptake and growth^12^. Additionally, researchers in Australia attribute a phase shift to tall algal turfs to a pulsed sedimentation event driven by rainfall^28^. In temperate communities, pulsed nutrient subsidies frequently drive macroalgal blooms^29,30^; if increased subsidies continue, rainfall events may potentially fuel macroalgal blooms on reefs. Thus, the ramifications of pulsed nutrients and sediments derived from extreme rainfall events may have persistent effects on coral reef benthic communities and are thus important to document and manage.

Suspended sediments were unprecedentedly high in nitrogen content, suggesting substantial transport of nitrogen from terrestrial to marine communities. Suspended sediments were red in color, implying terrestrial origin, and averaged 0.3% N. This is an order of magnitude higher than benthic sediment previously measured in Moorea at Gump Reef^31^ as well as benthic sediment measured in Jamaican tropical reefs^32^. Our data are also comparable to data from a study quantifying percent nitrogen in suspended sediments of 11 major rivers around the world, including the Fly River in Papao New Guinea, Tomalas Bay in California, USA, the Sacramento River, San Francisco Bay California USA, the Eel River California USA, Galveston Bay in Texas, USA, the Amazon River in Brazil, the Huanghe River in China, the Changjiang River in China, the MacKenzie River in Canada, the Mississippi River in Mississippi USA, and Chesapeake Bay in Maryland USA^33^. Percent N in these rivers ranged from <0.05 to ~0.6%. The percent N measured in this study exceeds 8 of these 11 rivers, is matched by the Mississippi (0.25–0.35%), and slightly exceeded by Tomales (max ~0.4%N) and the Chesapeake (max ~0.6%)^33^. Thus, our measurement of %N in the suspended sediment is high by a global standard, necessitating consideration of suspended material as a source of nutrients in nearshore communities, even on small, isolated tropical reefs.

While rainfall events often drive pulses of material transport via rivers and run-off, the magnitude of this transport likely is driven by anthropogenic activities. Previous research suggests the magnitude of transport from terrestrial to nearshore communities is increased by anthropogenic activities such as logging, agriculture, and urbanization^5–7,34^. Moorea sewage management is primarily septic and the catchment of Cooke’s Bay includes a goat farm as well as pineapple fields (*personal observation*); all three of these are likely sources of increased nutrient loading in the water column. These land use patterns likely increase both nutrient and sediment mobilization. The δN^15^ signature in our indicator alga highlight a shift to increased proportion of nitrogen from sewage sources^35^, likely driven by these land use patterns. Broadly, extreme rainfall events, combined with human activities that increase nutrients and mobility of sediments may increase pulsed transport of materials from terrestrial to marine communities, warranting continued research and management effort.

Global climate change likely will change global patterns of rainfall, making it crucial to document the magnitude, frequency, and duration of pulsed inputs and understand their impacts on nearshore community dynamics. The 2014 IPCC report states that extreme precipitation events over wet tropical regions very likely will become more intense and more frequent as global mean surface temperature increases. This may result in more pulsed events to most coral reefs via riverine transport in the near future as a result of global climate change^36^. For example, in Moorea, this will drive larger magnitude, lower frequency, and higher magnitude rainfall events that may result in more extreme transport events from terrestrial to nearshore communities. Thus, efforts should be made to document these extreme events and explore their consequences to nearshore communities in light of projected climate change.

In sum, extreme rainfall events were correlated with pulsed nutrient and sediment subsidies from terrestrial to nearshore communities, which may facilitate macroalgal proliferation on coral reefs. The magnitude of this transport is likely strongly driven by watershed land use patterns. Future climate change predictions suggest these extreme rainfall events will become more common in this system, and will likely favor macroalgal proliferation.

## Methods

### Study site

Moorea is one of the Society Islands in French Polynesia, and beginning in 2004, is the site of a Long Term Ecological Research Station (LTER). This is a volcanic island that formed 1.5–2 Ma and is typical of the islands in the South Pacific^37^. The island has a barrier reef 0.5–1.5 km offshore with a series of passes that allow oceanic flushing^38^. Moorea experiences a warm, wet season from November to April and a cooler, drier season between May and October.

Moorea has 2 large bays on the northern shore likely formed by river basins that filled during sea level rise in the Holocene (Fig. 1). These bays are 20–30 m deep and rimmed by fringing reefs. Both bays are close to oceanic passes and regularly flushed. During large storm events, to balance the increased water transport into the lagoon, these reef passes may transport surface waters out of the lagoon, onto the fore reef and offshore^38^.

### Rainfall

Between January 13^th^ and January 22^nd^, 2017, Moorea experienced an extreme rainfall event captured by routine monitoring. The LTER has collected routine weather data since 2006, with a weather station that measures rainfall every 5 minutes. This data undergoes quality control measures, and are publicly available^27^. We used this data set to estimate daily rainfall over the 10-day period of the storm.

### Sediment pulse

Following the storm, we determined the spatial and temporal extent of the sediment pulsed into the ocean from the river. To do this, we conducted a series of surveys where we measured secchi disk depth^39,40^. Secchi disks are the oldest optical instrument used to measure transparency in bodies of water (reviewed in^41^). Transparency is a measurement of light penetration influenced by suspended particles and phytoplankton. Interpretation of secchi depths can be limited by differences in light levels and angles as well as variation in observers. Additionally, secchi depths are only an estimate of suspended particulate matter (SPM), which can be overridden by pigments in the water column. Finally, secchi disk cannot discriminate between newly transported matter and resuspended matter. However, they are useful for detecting large differences in transparency. To determine the relationship between suspended sediment and distance from the river mouth, we took measurements from as close to the mouth of the bay as we could get (~0.25 km) to as far out the pass as possible (~3 km). We used a handheld GPS device to exactly record the samples for both sediment and nutrient samples, and calculated distance from the Pao Pau River mouth. We used a 45-cm wide white secchi disk to quantify secchi disk depths to estimate suspended sediments. Only one observer took all secchi disk measurements to minimize variability. We lowered the secchi vertically into the water column until it was no longer visible and recorded the depth and position (latitude and longitude). The last day of the storm (January 22, 2017), we surveyed in the morning (1000) and afternoon (1600) to capture rapid changes in suspended sediments. We then measured secchi disk depths 1, 2, 5, and 9 days after the storm. For each of these sampling times, we recorded 30 secchi disk depths (n = 180 total). To relate distance from the Pao Pao River mouth to suspended sediments, we fit logarithmic curves relating secchi disk depth to distance from the river mouth for each of our 6 sampling periods because we had an expectation that light would attenuate with depth.

To quantify the nutrients transported by the sediment, we collected water with suspended sediment following the storm. Water was collected on Gump Reef. Suspended sediments were allowed to settle, and sediments were collected and dried to a constant weight in an oven. Sediments were not filtered because the amount of suspended sediment clogged a handheld filter after less than 0.25 L of water was filtered. Then, sediments ground to a fine powder (n = 4) and sent to the UC Davis Stable Isotope Laboratory where samples were analyzed for percent nitrogen (N). To determine percent N, the UC Davis Analytical lab uses a PDZ Europa ANCA-GSL elemental analyzer and a PDZ Europa 20–20 isotope ratio mass spectrometer.

### Nutrient pulse

To characterize spatial and temporal patterns in concentrations of phosphate, nitrite + nitrate, ammonium, and for salinity following the rainfall event, we took water samples at multiple distances from the river mouth over multiple days. Water was sampled from the top meter of the water column at 5 distances from the mouth of the Pao Pao River, ranging from 0.3–2.6 km from the mouth of the river to determine spatial patterns (n = 3 for each distance). Water samples were taken at these 5 distances the last day of the storm, and then 1, 4, and 9 days after the storm passed to assess temporal patterns (n = 120 total). These water samples were frozen to −80 °C and remained frozen through transport until sent to an analytical lab for analysis. Water was analyzed at the UC Santa Barbara Analytical Lab, which used flow injection analysis to quantify phosphate (PO_4_), nitrate + nitrite (NO_3_ + NO_2_), and ammonia (NH_4_) concentrations. In flow injection analysis, a sample is injected into a carrier solution in a continuous flow system where it mixes with appropriate reagents before reaching a detector. Salinity was measured after samples were analyzed for nutrients with a refractometer. We compared our nutrient concentrations to routine monitoring data on phosphate and NO_3_ + NO_2_ water column concentrations from the MCR LTER^27^.

### Macroalgal tissue nutrients

To determine the transference of pulsed terrestrial nutrients to the marine benthic community, we collected *Padina boryana* from Gump Reef, located directly off Gump Research Station (Fig. 1), on days 1–10 after the storm to quantify patterns of uptake and incorporation of nutrients into macroalgal tissues over time. *P. boryana* was collected from the field just outside of the Gump Research Station, rinsed in freshwater, and dried (n = 3 per day). Macroalgal tissues were ground into a fine powder and sent to the UC Davis Stable Isotope Laboratory where samples were analyzed for both percent nitrogen (N) and δN^15^. Percent tissue N gives an estimate of uptake into tissues when measured over time. δN^15^ provides insight into nitrogen sources. Nitrogen from different sources has different N^14^:N^15^ ratios, which when compared to atmospheric N^14^:N^15^ ratios, can be used to calculate δN^15^ ^35^. This difference in ratios is due to bacterial processes affecting different nitrogen sources. Bacteria have a preference for N^14^ because it is lighter and easier to metabolize; thus, sewage has a higher ratio of N^15^ than other sources and a higher δN^15^ value. Thus, δN^15^ allows for assessment of nutrient origin where typically, atmospherically fixed N = 0, fertilizers = 0, raw sewage = 6–10, and oceanic sources are <3 (^35^). To determine δN^15^ and percent N, the UC Davis Analytical lab uses a PDZ Europa ANCA-GSL elemental analyzer and a PDZ Europa 20–20 isotope ratio mass spectrometer. Percent N is necessary to calculate δN^15^.

All work was approved by the Délégations Régionales à la Recherche et à la Technologie (D.R.R.T.) of the French Polynesia government via protocole d’accueil issued to CR Fong and all methods were carried out in accordance with relevant guidelines and regulations.

## Data Availability

The datasets generated for the current study are available from the corresponding author on reasonable request.

## References

[1] Fredston-Hermann A (2016). Where does river runoff matter for coastal marine conservation?. Frontiers in Marine Science.

[2] Hyndes GA (2014). Mechanisms and ecological role of carbon transfer within coastal seascapes. Biological Reviews.

[3] Halpern BS (2015). Spatial and temporal changes in cumulative human impacts on the world’s ocean. Nature Communications.

[4] Ban SS, Graham NA, Connolly SR (2014). Evidence for multiple stressor interactions and effects on coral reefs. Global Change Biology.

[5] Brodie JE (2012). Terrestrial pollutant runoff to the Great Barrier Reef: an update of issues, priorities and management responses. Marine Pollution Bulletin.

[6] Fabricius KE (2005). Effects of terrestrial runoff on the ecology of corals and coral reefs: review and synthesis. Marine pollution bulletin.

[7] Maina J (2013). Human deforestation outweighs future climate change impacts of sedimentation on coral reefs. Nature communications.

[8] Rabouille C, Mackenzie FT, Ver LM (2001). Influence of the human perturbation on carbon, nitrogen, and oxygen biogeochemical cycles in the global coastal ocean. Geochimica et Cosmochimica Acta.

[9] Vitousek PM (1997). Human alteration of the global nitrogen cycle: sources and consequences. Ecological Applications.

[10] Huang JC (2012). Stream discharge characteristics through urbanization gradient in Danshui River, Taiwan: perspectives from observation and simulation. Environmental Monitoring and Assessment.

[11] Yang LH, Bastow JL, Spence KO, Wright AN (2008). What can we learn from resource pulses. Ecology.

[12] den Haan J (2016). Nitrogen and phosphorus uptake rates of different species from a coral reef community after a nutrient pulse. Scientific reports.

[13] Fong CR, Fong P (2018). Nutrient fluctuations in marine systems: press versus pulse nutrient subsidies affect producer competition and diversity in estuaries and coral reefs. Estuaries and coasts.

[14] Briceño, H. O. & Boyer, J. N. Annual Report of theWater Quality Monitoring Project for the Water Quality Protection Program of the Florida Keys National Marine Sanctuary. SERC Research Reports. 113. http://digitalcommons.fiu.edu/sercrp/113 (2012).

[15] Moberg F, Folke C (1999). Ecological goods and services of coral reef ecosystems. Ecological economics.

[16] Odum HT, Odum EP (1955). Trophic structure and productivity of a windward coral reef community on Eniwetok Atoll. Ecological monographs.

[17] Fong, P. & Paul, V. J. Coral reef algae. In *Coral reefs: an ecosystem in transition* (pp. 241–272). Springer, Dordrecht (2011).

[18] Bainbridge ZT, Wolanski E, Álvarez-Romero JG, Lewis SE, Brodie JE (2012). Fine sediment and nutrient dynamics related to particle size and floc formation in a Burdekin River flood plume, Australia. Marine Pollution Bulletin.

[19] Schaffelke B, Carleton J, Skuza M, Zagorskis I, Furnas MJ (2012). Water quality in the inshore Great Barrier Reef lagoon: Implications for long-term monitoring and management. Marine Pollution Bulletin.

[20] Storlazzi CD, Field ME, Bothner MH, Presto MK, Draut AE (2009). Sedimentation processes in a coral reef embayment: Hanalei Bay, Kauai. Marine Geology.

[21] Risk MJ (2014). Assessing the effects of sediments and nutrients on coral reefs. Current Opinion in Environmental Sustainability.

[22] Devlin MJ (2012). Mapping the pollutants in surface riverine flood plume waters in the Great Barrier Reef, Australia. Marine pollution bulletin.

[23] Ringuet S, Mackenzie FT (2005). Controls on nutrient and phytoplankton dynamics during normal flow and storm runoff conditions, southern Kaneohe Bay, Hawaii. Estuaries.

[24] Charpy-Roubaud CJ, Charpy L, Cremoux JL (1990). Nutrient budget of the lagoonal waters in an open central South Pacific atoll (Tikehau, Tuamotu, French Polynesia). Marine Biology.

[25] Bellwood DR, Hughes TP, Folke C, Nyström M (2004). Confronting the coral reef crisis. Nature.

[26] Fong CR, Bittick SJ, Fong P (2018). Simultaneous synergist, antagonistic and additive interactions between multiple local stressors all degrade algal turf communities on coral reefs. Journal of Ecology.

[27] Alldredge, A. Moorea Coral Reef LTER. MCR LTER: Coral Reef: Water Column: Nutrients, ongoing since 2005. knb-lter-mcr.1034.9 (2019)

[28] Goatley CH, Bonaldo RM, Fox RJ, Bellwood DR (2016). Sediments and herbivory as sensitive indicators of coral reef degradation. Ecology and Society..

[29] Cohen RA, Fong P (2004). Nitrogen uptake and assimilation in Enteromorpha intestinalis (L.) Link (Chlorophyta): using 15N to determine preference during simultaneous pulses of nitrate and ammonium. Journal of experimental marine biology and ecology.

[30] Kennison RL, Kamer K, Fong P (2011). Rapid nitrate uptake rates and large short‐term storage capacities may explain why opportunistic green macroalgae dominate shallow eutrophic estuaries 1. Journal of Phycology.

[31] Clausing RJ (2014). Effects of sediment depth on algal turf height are mediated by interactions with fish herbivory on a fringing reef. Marine Ecology Progress Series.

[32] Mills MM, Sebens KP (2004). Ingestion and assimilation of nitrogen from benthic sediments by three species of coral. Marine Biology.

[33] Mayer LM (1998). Importance of suspended participates in riverine delivery of bioavailable nitrogen to coastal zones. Global Biogeochemical Cycles.

[34] Townsend‐Small A (2013). Increasing summer river discharge in southern California, USA, linked to urbanization. Geophysical Research Letters.

[35] Costanzo SD, O’donohue MJ, Dennison WC, Loneragan NR, Thomas M (2001). A new approach for detecting and mapping sewage impacts. Marine Pollution Bulletin.

[36] IPCC, Climate change 2014: Synthesis report. In Contribution of working groups I, II and III to the fifth assessment report of the intergovernmental panel on climate change, ed. Core Writing Team, R.K. Pachauri & L.A. Meyer. Geneva: IPCC (2014).

[37] Neall VE, Trewick SA (2008). The age and origin of the Pacific islands: a geological overview. Philosophical Transactions of the Royal Society B: Biological Sciences.

[38] Leichter JJ (2013). Biological and physical interactions on a tropical island coral reef: transport and retention processes on Moorea, French Polynesia. Oceanography.

[39] Holmes RW (1970). The secchi disk in turbid coastal waters 1. Limnology and Oceanography.

[40] Kulshreshtha A, Shanmugam P (2015). Estimation of Secchi transparency in turbid coastal waters. Aquatic Procedia.

[41] Lee Z (2015). Secchi disk depth: A new theory and mechanistic model for underwater visibility. Remote sensing of environment.

